# Guidance for updating clinical practice guidelines: a systematic review of methodological handbooks

**DOI:** 10.1186/1748-5908-9-3

**Published:** 2014-01-02

**Authors:** Robin WM Vernooij, Andrea Juliana Sanabria, Ivan Solà, Pablo Alonso-Coello, Laura Martínez García

**Affiliations:** 1 Iberoamerican Cochrane Centre, Institute of Biomedical Research (IIB Sant Pau), C/ Sant Antoni Maria Claret 167, Barcelona 08025, Spain; 2 Department of Health Sciences, Faculty of Earth and Life Sciences, VU University, Amsterdam, The Netherlands

**Keywords:** Clinical practice guidelines, Evidence-based medicine, Handbooks, Methodology, Systematic review

## Abstract

**Background:**

Updating clinical practice guidelines (CPGs) is a crucial process for maintaining the validity of recommendations. Methodological handbooks should provide guidance on both developing and updating CPGs. However, little is known about the updating guidance provided by these handbooks.

**Methods:**

We conducted a systematic review to identify and describe the updating guidance provided by CPG methodological handbooks and included handbooks that provide updating guidance for CPGs. We searched in the Guidelines International Network library, US National Guidelines Clearinghouse and MEDLINE (PubMed) from 1966 to September 2013. Two authors independently selected the handbooks and extracted the data. We used descriptive statistics to analyze the extracted data and conducted a narrative synthesis.

**Results:**

We included 35 handbooks. Most handbooks (97.1%) focus mainly on developing CPGs, including variable degrees of information about updating. Guidance on identifying new evidence and the methodology of assessing the need for an update is described in 11 (31.4%) and eight handbooks (22.8%), respectively. The period of time between two updates is described in 25 handbooks (71.4%), two to three years being the most frequent (40.0%). The majority of handbooks do not provide guidance for the literature search, evidence selection, assessment, synthesis, and external review of the updating process.

**Conclusions:**

Guidance for updating CPGs is poorly described in methodological handbooks. This guidance should be more rigorous and explicit. This could lead to a more optimal updating process, and, ultimately to valid trustworthy guidelines.

## Background

Clinical practice guidelines (CPGs) intend to patient care by providing recommendations about the benefits and downsides of best practice in healthcare [1]. If adequately implemented, CPGs have the potential of reducing variability and translating scientific research into clinical practice and consequently improve the quality and safety of healthcare [2-4].

However, scientific knowledge is in constant change; therefore CPGs need to be updated regularly to maintain validity [5]. The obsolescence of a CPG might occur because of new scientific research, including the development of new technologies in treatment and diagnosis alternatives, economic differences, or changes in values and preferences [6,7]. Generally, an updating process consists of three components: the identification of new evidence, the assessment of the need to update, and the formulation of new or modified recommendations [5,8-11]. Some authors suggest that an update is generally required after three to five years; however, little research has been undertaken so far [8,12,13].

Several institutions responsible for developing CPGs drafted their own methodological handbooks including methodology for developing and updating their CPGs. Some of these handbooks are very influential and often used in smaller organizations [6,14]. Even though the methodology developed greatly over the last years, the quality of CPGs is lagging behind [1,15,16]. A lack of compliance with state of the art methodology for developing CPGs has been found, and hence the methodological quality of CPGs remained very similar over the last two decades [17,18]. Little is known about the guidance for updating CPGs included in these handbooks [19,20]. Therefore, we systematically reviewed CPGs methodological handbooks to identify and describe the methodological guidance about updating.

## Methods

### Search strategy

We conducted a systematic search in September 2013 in MEDLINE (via PubMed, from 1966 onwards), using a combination of free text terms (Clinical Practice Guidelines, Clinical Guidelines, Guidelines, Methodolog*, Handbook*). The search strategy is available as supplementary data (Additional file 1). In addition, we searched: the database of the Guidelines International Network (http://www.g-i-n.net); the US National Guidelines Clearinghouse database (http://www.guidelines.gov); and the website of institutions that reported to use a methodological handbook in a previous international survey conducted by our group [12]. If necessary, we contacted organizations to obtain the handbooks.

### Eligibility criteria

We included methodological handbooks that provide guidance on the updating process of CPGs. Handbooks that exclusively report methodologies for developing *de novo* guidelines were excluded. We included handbooks regardless of their language or publication status. When necessary, the handbook was translated.

### Study selection

Two authors (RV, AJS) independently selected potential handbooks by reviewing titles and abstracts, and finally full text for a more detailed evaluation. Disagreements were initially resolved by consensus, and if necessary, with the help of a third author (PA-C).

### Data extraction

Based on our previous experiences concerning updating, including an international survey [12] a systematic review [8] and additional relevant literature [5,6,9-11,14] we developed, reviewed, and piloted iteratively a case report form (CRF). After consensus, the following items are included in the CRF: characteristics of the handbook and institution, group responsible for updating CPGs, strategy for identifying new evidence, methodology for assessing the need for an update, methods for the literature search, evidence selection, evidence assessment, evidence synthesis, external review, and for the edition and dissemination of the updated CPG. The CRF can be made available upon request.

Two authors (RV, AJS) extracted independently the data of the handbooks accepted for inclusion. Disagreements were initially resolved by consensus, and if necessary, with the help of a third author (PA-C). While extracting the data, we considered a strategy to be specific if the handbook included a detailed methodology, enabling the reader to conduct the suggested strategy. We considered a non-specific strategy if not enough methodological guidance is provided to facilitate an adequate approach.

### Data analysis

We used descriptive statistics to analyze the extracted data. We calculated absolute frequencies and proportions for all items. In addition, we conducted a narrative synthesis. Data analysis was performed using SPSS statistical software, version 18.0 (SPSS INC., Chicago, IL, USA). By consensus of two authors (RV, AJS), we collected relevant quotations within the themes included in the handbooks and provide these in the free text area.

## Results

### Handbooks selection

**Figure 1:**
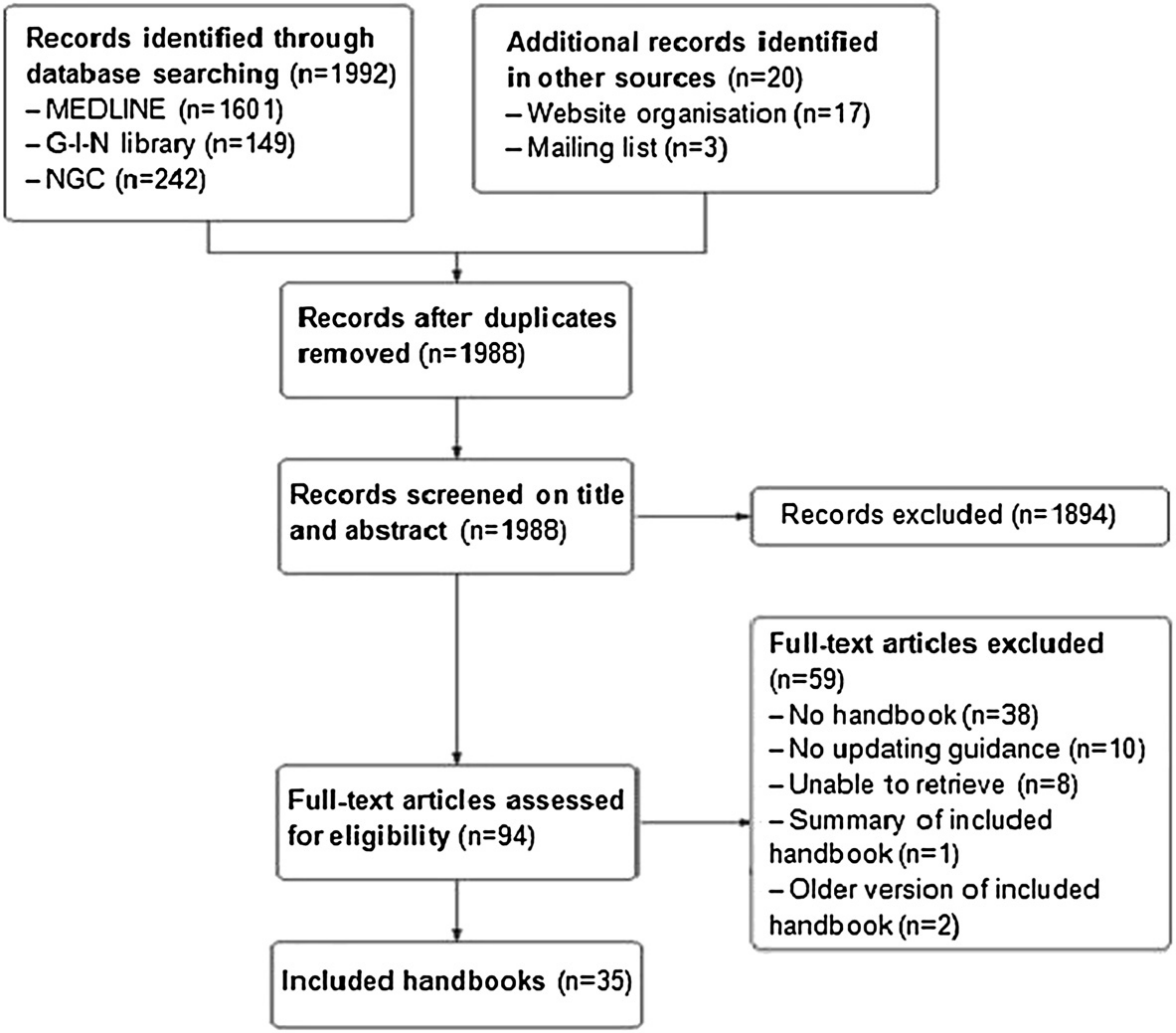
Flow chart of the screening literature process.

We screened the titles and abstracts of 1,992 references (Figure 1). We selected 94 articles for full-text review. Thirty-eight articles were excluded because they were not methodological handbooks. Additionally, ten handbooks were excluded because they exclusively focused on developing *de novo* CPGs. We could not locate eight articles and one article was a summary of an included handbook. Two handbooks were excluded because a more recent version was included. Additional file 2 provides an overview of the excluded documents. Finally, we included thirty-five handbooks (Additional file 3) [5,6,14,21-52].

### Handbooks characteristics

In total, 48.6% of the included handbooks are developed by institutions based in Europe [5,6,14,21-34] mostly being public institutions (57.1%) (Table 1) [5,6,14,22-26,28,31,35-43]. One handbook (2.9%) addresses specifically the methodology of updating CPGs [5]; the others (97.1%) focus mainly on developing *de novo* CPGs, and include variable degrees of information about updating [6,14,21-52]. Fourteen handbooks (40.0%) are published between 2005 and 2010 [5,21,23,26,30,32,34,39],[40,43,44,46,48,50].

**Table 1:** Characteristics of institutions and handbooks

**Institution characteristics**		
	**n**	**(%)**
**Continent**		
Europe	17	48.6
North America	12	34.3
Oceania	4	11.4
International	2	5.7
**Type of organization**		
Public institution	20	57.1
Scientific society	9	25.7
Private organism	3	8.6
Other (Federal institute, NGO)	3	8.6
**Number of years developing guidelines**		
≤10 years	10	28.6
10 – 20 years	19	54.3
>20 years	6	17.1
**Number of guidelines published**		
≤5 per year	22	62.9
>5 per year	8	22.9
Unknown	5	14.3
**Handbook characteristics**		
**Type of handbook**		
Development CPG handbook	34	97.1
Update CPG handbook	1	2.9
**Publication date**		
Before the year 2004	8	22.9
Between 2005 – 2010	14	40.0
Between 2011 – 2013	8	22.9
Unknown	5	14.3

### Updating group

The persons responsible for updating the CPG are specified in twelve handbooks (34.3%). Seven handbooks (20.0%) state that the updating group should have a similar structure to the group that contributed to developing the CPG [6,14,23,30,37,44,45]. Four handbooks (11.4%) state that the group, responsible for updating the CPG, should be tailored to the new scope of the guideline [5,38,39,41].

### Time between updates

Twenty-five (71.4%) of the included handbooks recommend a time frame between publishing a CPG and commencing an updating process (Table 2), with two to three years being the most frequently recommended (40.0%) [5,6,14,22,27,28,30-32,37],[39,41,45,46]. Furthermore, three handbooks (8.6%) suggest a time frame of less than one year [33,34,44], and eight handbooks (22.9%) include a four to five year time frame [24,36,38,42,43,47-49].

**Table 2:** Guidance reported in the included handbooks

**Group responsible for updating CPG**		
	**n**	**(%)**
**Are the participants in the updating group specified?**		
Yes	12	34.3
No	23	65.7
**What members do the updating group consist of?**		
Similar to the development team	7	20.0
Updating group specifically defined	4	11.4
Not defined	24	68.6
**Identification of new evidence**		
**Time frame for updating**		
≤1 year	3	8.6
2-3 years	14	40.0
4-5 years	8	22.9
No specific time frame indicated	10	28.6
**Identification of new evidence**		
Specific strategy	9	25.7
Non specific strategy	2	5.7
Not defined	24	68.6
**Assessment of the need for an update**		
**Assessment of the need for an update**		
Specific strategy	8	22.8
Not defined	27	77.1
**Updating strategy**		
**Distinction between different updates (partial / full)**		
Yes	8	22.9
No	27	77.1
**Literature search**		
Specific strategy	11	31.4
Similar to the development process	6	17.1
No strategy defined	18	51.4
**Evidence selection**		
Specific strategy	3	8.6
Similar to the development process	8	22.9
Not defined	24	68.6
**Evidence assessment**		
Specific strategy	5	14.3
Similar to the development process	8	22.9
Not defined	22	62.9
**Evidence synthesis**		
Specific strategy	3	8.6
Similar to the development process	5	14.3
Not defined	27	77.1
**External review**		
Specific strategy	5	14.3
Similar to development process	6	17.1
Non specific strategy	2	5.7
Not defined	22	62.9
**Edition and dissemination**		
**Indication of changes**		
Specific strategy	5	14.3
Not defined	30	85.7
**Dissemination of the updated CPG**		
Specific strategy	3	8.6
Not defined	32	91.4

### Identification of new relevant evidence

**Figure 2:**
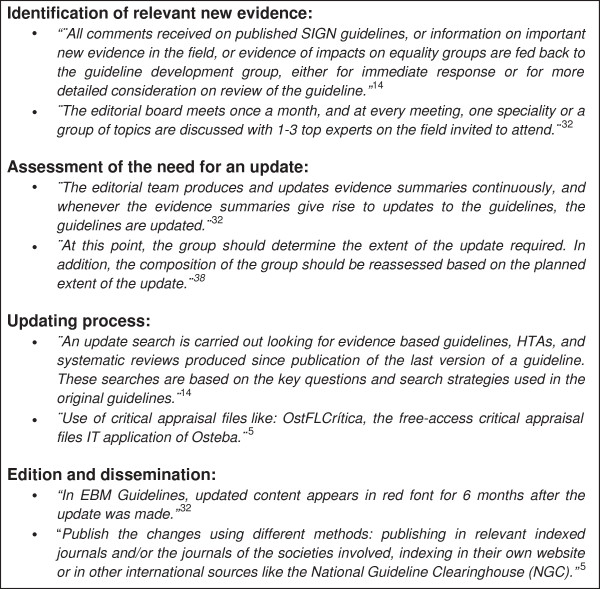
Box of relevant comments.

Eleven handbooks (31.4%) provide guidance on how to identify new relevant evidence. Of these eleven handbooks, six (17.1%) suggest using opinions or experiences from experts, users, or members of the original development group for identifying new relevant evidence [5,14,23,37,43,46]. Five handbooks (14.3%) provide guidance on conducting limited searches to identify new relevant evidence [5,37-39,47]. Furthermore, two handbooks (5.7%) propose the editorial board to have periodic meetings to discuss topics with experts [32,33]. One handbook (2.9%) suggests collecting alerts to identify newly published articles [5]. Externally reviewing the CPG by experts, who were not involved in developing the CPGs, is recommended by one handbook (2.9%) [47]. Two other handbooks (5.7%) provide a ‘non-specific strategy’ and only emphasize the importance of identifying new relevant evidence (Table 2) [23,28]. Figure 2 shows examples of relevant passages included in the handbooks.

### Assessment of the need for an update

The methodology of assessing the need for an update is described in eight handbooks (22.8%). Six of them (17.1%) give guidance on how to assess the importance and relevance of the new evidence, the disagreement between the new evidence and current recommendations, and whether the new knowledge is not yet included [5,6,23,38,43,49]. Two handbooks (5.7%) recommend expert judgment to assess the need for an update [38,40]. Producing and regularly updating evidence summaries and assessing the need for an update with these summaries are described in one handbook (2.9%) (Figure 2) [32].

### Updating recommendations

Eight handbooks (22.9%) provide guidance on what type of update is required in specific situations, by making a distinction between partial or full updates (Table 2) [5,6,14,33,37,38,43,44].

Guidance for conducting a literature search strategy is included in seventeen handbooks (48.6%). Eight of them (22.8%) include guidance to adjust the original search strategy [5,6,14,24,26,27,37,43]. Four handbooks (11.4%) provide guidance on what kind of evidence to search for, including evidence based guidelines, health technology assessments, systematic reviews, and randomized controlled trials [14,27,38,41]. Two handbooks (5.7%) recommend to include a medical librarian or research officer in the team to conduct the literature searches [41,48]. Using multiple databases, *e.g.*, MEDLINE and Cochrane Library, in the search strategy is recommended by two handbooks (5.7%) [41,43]. Furthermore, six handbooks (17.1%) suggest using the original strategy used for the development of the original guideline (Table 2, Figure 2) [23,28,34,40,44,50].

Eleven handbooks (31.4%) provide guidance for selecting adequate evidence in the updating process. Three handbooks (8.6%) provide specific guidance on how to discard irrelevant information [5,14,44]. Eight handbooks (22.9%) refer the reader to the development process for guidance on evidence selection [6,27,28,34,37,38,48,50].

Guidance for evidence assessment is provided in thirteen handbooks (37.1%). The assessment of the available evidence on the consistency, directness, validity or reliability is described in four handbooks (11.4%) [14,37,43,48]. Using critical appraisal frameworks, like OstFLCritica, is recommended in one handbook (2.9%) (Figure 2) [5]. Eight handbooks (22.9%) recommend the same original development strategy [6,23,27,28,34,38,44,50].

Similarly, guidance for the evidence synthesis is described in eight handbooks (22.9%). Three handbooks (8.6%) recommend producing evidence tables including the characteristics of included studies, quality of randomized trials, results for continuous outcomes, and results for dichotomous outcomes [14,43,48]. Moreover, five handbooks (14.3%) direct the reader to the section with guidance for evidence synthesis used for developing *de novo* CPGs [5,6,34,44,50].

Guidance for an external review of the updated CPG is described in thirteen handbooks (37.1%). Five handbooks (14.3%) describe the process of external reviewing the updated CPG by multiple external reviewers [37,43,45,47,48]. Furthermore, two handbooks (5.7%) provides ‘non-specific guidance’ for conducting an external review of the updated CPG [28,38]. Six handbooks (17.1%) refer to the guidance described in the section of developing *de novo* CPGs [5,6,27,34,44,50].

### Edition and dissemination

Two handbooks (5.7%) suggest to post a notification on the website of the institution whenever the need for an update is confirmed [28,29]. Five handbooks (14.3%) include a specific strategy for indicating the changes made in the update (Table 2, Figure 2). These handbooks recommended actions to identify the main changes in the update without any difficulty, including a table of updated evidence, summary reports, or highlight the updated parts in the text with a red font [5,32,33,37,47].

Three handbooks (8.6%) provide guidance on how to publish and disseminate the updated CPG. All three of them include methods to disseminate the updated CPG as widely as possible by publishing in relevant indexed journals [5], disseminate within the patient organization of the specific disease [48], or working together with public and private partners to reach specific groups and individuals [43].

## Discussion

We systematically reviewed 35 methodological handbooks that provide some type of guidance on the updating process of CPGs. Our results show that overall the updating guidance is poorly described. Crucial elements in identifying new evidence, the assessment for the need for an update and the updating strategy itself, are generally lacking or include solely a reference to the development process. Our findings resonate with previous findings that suggest that there is a need for rigorous international guidance for updating CPGs [8,14].

**Figure 3:**
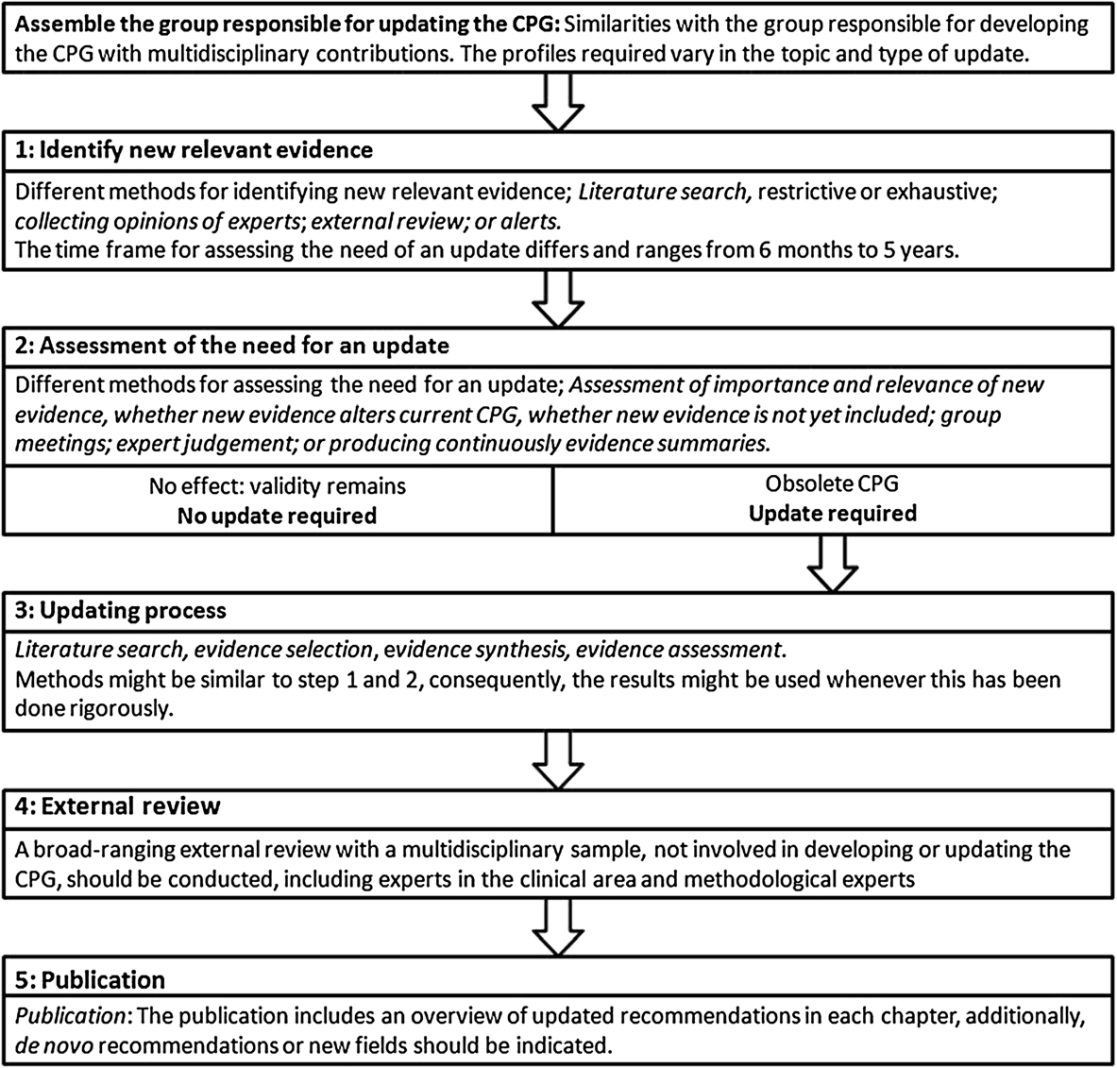
The updating process of CPGs.

Figure 3 summarizes an updating process framework for CPGs based on a previous systematic review from our group and the results of the present study [8]. The process of updating a CPG starts with assembling a group responsible for updating the CPG. However, we found that the majority of the institutions (65.7%) do not include any information about this first step. There is no clear consensus on who should participate in an updating process and, consequently different organizations use different strategies, depending on the characteristics of the organization and type of update. An updating working group, should consist of individuals with a background in methodology and experts in the field of interest, just as the original guideline group [5]. New developments in the clinical area, such as new technologies, might require including additional members with different expertise.

The actual updating process starts with identifying new relevant evidence. Currently, the period between the last publication of the CPG and starting the updating process (time frame) is frequently determined at the time of publication. The majority of the handbooks (62.9%) include a fixed time frame from two to five years, consistent with the results of previous research by Shekelle et al. [13]. This study including a sample of 17 guidelines, estimated that approximately one-half of the CPGs will be outdated after 5.8 years (95% CI: 5.0 – 6.6), and 10% are obsolete after 3.6 years (95% CI: 2.6 – 4.6) [13]. However, these average estimates can be misleading as CPG deteriorating speed is highly topic-specific, with some fields with rapid developments requiring more frequent surveillance for new evidence than others. Suboptimal time frames are likely to result in guidelines becoming obsolete or inefficient use of resources.

After identifying new relevant evidence, an assessment of the effect of this new evidence should be conducted, determining the need for an update [5,9-11]. We believe that this process is best conceptualized as a two-stage process because these are two independent stages with identifying possible new relevant evidence as first step, and, subsequently, deciding whether the identified evidence this evidence alters the validity of the current recommendations as second step. However, at the moment, formal explicit procedures for assessing the need for an update are not available, with most of the included handbooks (77.1%) not providing explicit methods for assessing the need for an update.

When the need for an update is confirmed, the new evidence has to be incorporated in the current recommendations. However, less than one-half of the included handbooks state specific methods for this process. Previous studies suggest a model of assessing the need for an update using expert opinion, focused literature reviews, and consensus meeting [11,13]. A reference to the development process, often included in the evaluated handbooks, is not enough because the aim of any update should be to incorporate new evidence in the context of previous recommendations. More specific methods should be included in the handbooks.

A further problem is that several institutions use different terminology and consequently bring further confusion. Some institutions use the term ‘monitoring’ for the identification of new evidence and assessment of the need for an update, often within an abridged time frame [5,14,32,33,37,43,44,52]. In addition, the term ‘dynamic updating’ and ‘living guideline’ is used indistinctively, suggesting that CPGs are updated promptly and are always up-to-date [14,40,46]. Nevertheless, none of these handbooks provide guidance for conducting these processes and there is no consensus on when a guideline starts being dynamic or can be considered as a living guideline (Figure 3). We suggest avoiding these terms because it solely reflects the aspect of time between two versions. In Figure 3, we include a proposal regarding consistent terminology. Further research and consensus is needed in the international community about coherent terminology.

Our study is, as far as we know, the first study to examine the guidance about the updating process provided by CPG methodological handbooks. Our work has several strengths. We conducted a systematic and exhaustive search that included main databases, clearinghouses, and several institutions identified by a previous survey [12]. In addition, we contacted several organizations to retrieve non-published handbooks; therefore we believe that we included most of the existing handbooks. We independently performed eligibility and data extraction with a CRF developed and piloted by a group with extensive experience in the field.

Our study, however, might be subject to some limitations. It is possible that, after our extensive literature search, we did not identify all available handbooks because some are not indexed nor published, and only used for in-house purposes. However, unpublished handbooks are likely to be of lower quality. If this is the case, it would imply that we overestimated the quality of the updating guidance, further strengthening our conclusions. Finally, the reported methods in handbooks might not reflect the actual updating in CPGs. However, we believe that this is unlikely given previous results of our international survey with CPG developers [12].

## Conclusion

Our work shows that updating guidance included in CPGs methodological handbooks is overall of poor quality. CPGs developers should provide more explicit and rigorous guidance and standardize the terminology used. This could, consequently, lead to a more optimal updating process of CPGs, and ultimately, to valid trustworthy guidelines.

## Abbreviations

CPGs: Clinical practice guidelines; CRF: Case report form.

## Competing interests

PA-C is an author of one of the included handbooks. For this reason, other authors completed data extraction for this handbook.

## Authors’ contribution

Conceiving the review: PA-C, LM. Design of the study: PA-C, LM, RV, AJS. Undertaking searches: IS, RV. Screening and extracting data: RV, AJS. Writing the review: RV, AJS, PA-C. Comment and editing of review drafts: all authors. All authors read and approved the final manuscript.

## Authors’ information

RV is a doctoral candidate at the Paediatrics, Obstetrics and Gynaecology and Preventive Medicine Department, Universitat Autònoma de Barcelona, Barcelona, Spain.

## Supplementary Material

Additional file 1 Search strategy (September 16, 2013). Click here for file

Additional file 2 List of excluded studies after full-text evaluation [in alphabetic order]. Click here for file

Additional file 3 Included handbooks [ordered by organisation]. Click here for file
