# Supplementary material for: Guidance for updating clinical practice guidelines: a systematic review of methodological handbooks
Source: Implement Sci. 2014 Jan 2;9:3. doi: 10.1186/1748-5908-9-3 (PMC3904688; doi:10.1186/1748-5908-9-3)
Supplement: Additional file 1 — Search strategy (September 16, 2013). [file 1748-5908-9-3-S1.pdf]

## Additional file 1: Search strategy (September 16, 2013)

### MEDLINE (PubMed)

| | | |
|---|---|---|
| #1 | Clinical Practice Guideline*[tw] | 6887 |
| #2 | Clinical guideline*[tiab] | 6397 |
| #3 | Guideline*[ti] | 47004 |
| #4 | (#1 OR #2) OR #3 | 53798 |
| #5 | Methodolog* [tiab] | 206771 |
| #6 | Handbook* [tiab] | 1902 |
| #7 | #5 OR #6 | 208439 |
| #8 | ((#4) AND #7) | 1935 |
| #9 | Practice Guideline[pt] OR Guideline[pt] | 24053 |
| #10 | (#8) NOT #9 | **1601** |
