# Supplementary material for: Guidance for updating clinical practice guidelines: a systematic review of methodological handbooks
Source: Implement Sci. 2014 Jan 2;9:3. doi: 10.1186/1748-5908-9-3 (PMC3904688; doi:10.1186/1748-5908-9-3)
Supplement: Additional file 2 — List of excluded studies after full-text evaluation [in alphabetic order]. [file 1748-5908-9-3-S2.pdf]

Additional File 2: List of excluded studies after full-text evaluation [in alphabetic order]

Number	Reference	Reason for exclusion
1	[No authors listed] **Methodology of guideline development.** *Neurosurgery* 2002,**50**(3):S2-6.	No handbook
2	Al-Benna S. **Construction and use of wound care guidelines: an overview.** *Ostomy Wound Manage* 2012,**58**(8):37-47.	Unable to retrieve
3	Agency for Healthcare Research and Quality. **Evidence-based Practice Program of AHRQ.** Rockville: Agency for Healthcare Research and Quality 2008.	No handbook
4	Agency for Quality in Dentistry. **Methodische Empfehlungen für Leitlinien in der Zahn-, Mund- und Kieferheilkunde (Leitlinien für Leitlinien).** Berlin: Agency for Quality in Dentistry 1999.	No handbook
5	American College of Chest Physicians. **Methodology for antithrombotic and thrombolytic therapy guideline development - American College of Chest Physicians evidence based clinical practice guidelines (8th edition).** Northbrook: American College of Chest Physicians 2008.	No handbook
6	Amerling R, Winchester JF, Ronco C. **Guidelines for guidelines.** *Blood Purif* 2007,**25**(1):36-8.	No handbook
7	Association of Scientific Medical Societies. **Erarbeitung von Leitlinien für Diagnostik und Therapie. Methodische Empfehlungen (AWMF).** Düsseldorf: Association of Scientific Medical Societies 2004.	No handbook
8	Association of Scientific Medical Societies. **Methodology for the development of the ESPEN Guidelines on Enteral Nutrition. S3-LL (DGEM).** Düsseldorf: Association of Scientific Medical Societies 2012.	No handbook
9	Basque Office for Health Technology Assessment. **Informe sobre la elaboración dela Guía GuNFT. Guía para la No Financiación de Tecnologáas sanitarias ya existents en los sistemas sanitarios.** San Sebastián: Basque Office for Health Technology Assessment 2009.	No handbook
10	Belgian Centre for Evidence-Based Medicine. **CEBAM Guide to guidelines.** Leuven: Belgian Centre for Evidence-Based Medicine 2005.	No handbook
11	Berlin Chamber of Physicians. **Richtlinien, Leitlinien, Empfehlungen der Bundesärztekammer – übersicht.** Berlin: Berlin Chamber of Physicians 2007.	No handbook
12	Bowen JM, Elad S, Hutchins RD, Lalla RV. **Mucositis Study Group of the Multinational Association of Supportive Care in Cancer/International Society of Oral Oncology (MASCC/ISOO). Methodology for the MASCC/ISOO Mucositis Clinical Practice Guidelines Update.** *Support Care Cancer* 2013,**21**(1):303-8.	No handbook
13	Burgers JS, van Everdingen JJ. **[Evidence-based guideline development in the Netherlands: the EBRO platform]**. *Ned Tijdschr Geneeskd* 2004,**148**(42):2057-9.	No updating guidance

14	Catalan Agency for Health Information, Assessment and Quality. **OncoGuies: guies de practica clínica en cancer de Catalunya.** Barcelona: Catalan Agency for Health Information, Assessment and Quality 2006.	No handbook
15	Clinical Epidemiology Centre. **Recomandations pour la préparation: de Recommandations de Pratique Clinique (´guidelines´).** Lausanne: Clinical Epidemiology Centre 2003.	No updating guidance
16	Duodecim Finnish Medical Society. **Current Care Guidelines – Programme Information.** Helsinki: Duodecim Finnish Medical Society 2004.	Unable to retrieve
17	Dutch Institute for Healthcare Improvement. **Development of CBO-Guidelines – Manual.** Utrecht: Dutch Institute for Healthcare Improvement 2003.	Summary of included full-text handbook
18	Dutch Institute for Healthcare Improvement. **Evidence-based richtlijnontwikkeling: Een leidraad voor de praktijk.** Utrecht: Dutch Institute for Healthcare Improvement 2004.	No handbook
19	Eccles M, Clapp Z, Grimshaw J, Adams PC, Higgins B, Purves I, Russell I. **Developing valid guidelines: methodological and procedural issues from the North of England Evidence Based Guideline Development Project.** *Qual Health Care* 1996,**5**(1):44-50.	No updating guidance
20	Echer IC. **Elaboração de manuais de orientação para o cuidado em saúde.** *Rev Lat Am Enfermagem* 2005,**13**(5):754-7.	No handbook
21	Figlin RA, Gilden R, Taylor C. **Methodology of the Practice Guidelines.** *Cancer J Sci Am* 1996,**2**(3A):S4.	Unable to retrieve
22	French National Authority for Health. **Guide des methods et outils de gestion des risques en établissement de santé. Note de cadrage. Guide méthodologique.** Saint-Denis La Plaine Cedex: French National Authority for Health 2011.	No handbook
23	French National Authority for Health. **Les conferences de consensus – base méthodologique pour leur realisation en France.** Saint-Denis La Plaine Cedex: French National Authority for Health 1999.	Unable to retrieve
24	French National Authority for Health. **Principes méthodologique pour la gestion des risques en établissement de santé. Guide méthodologique.** Saint-Denis La Plaine Cedex: French National Authority for Health 2003.	No handbook
25	French National Authority for Health. **Recommandations par consensus formalisé (RCF). Méthode d´elaboration de recommandations de bonne pratique. Guide méthodologique.** Saint-Denis La Plaine Cedex: French National Authority for Health 2010.	No handbook
26	Gaebel W, Falkai P. **Practice guidelines in psychiatry. Methodology and status of guideline development.** *Nervenarzt* 1996,**67**(2):179-81.	Unable to retrieve
27	German Agency for Quality in Medicine. **Allgemeiner Leitlinienreport (Leitliniengruppe Hessen).** Berlin: German Agency for Quality in Medicine 2009.	No handbook

28	German Agency for Quality in Medicine. **DM-CPG programme (German national disease management guidelines programme).** Berlin: German Agency for Quality in Medicine 2006.	No handbook
29	German Agency for Quality in Medicine. **Methoden-Report 4. Aufl.** Berlin: German Agency for Quality in Medicine 2010.	No handbook
30	Häuser W, Bernardy K, Wang H, Kopp I. **Methodenreport zur Erstellung der Leitlinie.** *Schmerz* 2012,**26**(3):232-46.	No handbook
31	Institute for Quality and Efficiency in Health Care. **CoCanADAPTE Manual: Processes and Toolkit.** Cologne: Institute for Quality and Efficiency in Health Care 2009.	No handbook
32	Italian National Institute of Health. **La produzione di raccomandazioni cliniche con il metodo GRADE. L´esperienza sui farmaci oncologici. Linea guida regionali Emilia-Romagna.** Rome: Italian National Institue of Health 2009.	No handbook
33	Kingston M, Radcliffe K, Daniels D, FitzGerald M, Lazaro N, McCarthy G, Sullivan A. **Clinical Effectiveness Group of British Association for Sexual Health and HIV. British Association for Sexual Health and HIV: framework for guideline development and assessment.** *Int J STD AIDS* 2010,**21**(7):453-6.	No updating guidance
34	Kopp I. **Grundsätze der Erstellung und Handhabung von Leitlinien.** *Radiologe* 2008,**48**(11):1015-6,1018-21.	No handbook
35	Lang ES, Spaite DW, Oliver ZJ, Gotschall CS, Swor RA, Dawson DE, Hunt RC. **A national model for developing, implementing, and evaluating evidence-based guidelines for prehospital care.** Acad Emerg Med. 2012,**19**(2):201-9.	No handbook
36	Loblaw DA, Prestrud AA, Somerfield MR, Oliver TK, Brouwers MC, Nam RK, Lyman GH, Basch E, Americal Society of Clinical Oncology Clinical Practice Guidelines. **American Society of Clinical Oncology Clinical Practice Guidelines: formal systematic review-based consensus methodology.** *J Clin Oncol* 2012,**30**(25):3136-40.	No updating guidance
37	Madan I, Paterson S, Harling K. **Methodology for the development of NHS Plus evidence-based guidelines.** *Occup Med (Lond)* 2007,**57**(5):304-7.	No updating guidance
38	Magit AE, Stool SE. **Clinical guideline development for otitis media: a report on methodology.** *Otolaryngol Head Neck Surg* 1993,**109**(3 Pt 1):478-81.	No handbook
39	Manno C, Strippoli GF, Cianciaruso B, Cagnoli L, Cancarini G, Messa P, Segoloni GP, Stratta P, Triolo G, Schena FP, Coordinatore del Gruppo di Studio di Evidence Based Nephrology, Steering Committee. **[The Italian Society of Nephrology Guidelines (3rd Edition): principles and methods]**. *G Ital Nefrol* 2006,**23**(2):173-81.	No updating guidance
40	McCormick KA, Fleming B. **Clinical practice guidelines. The Agency for Health Care Policy and Research fosters the development of evidence-based guidelines.** *Health Prog* 1992,**73**(10):30-4.	Unable to retrieve

41	McCrory DC, Colice GL, Lewis SZ, Alberts WM, Parker S. **Overview of methodology for lung cancer evidence review and guideline development.** *Chest* 2003,**123**(1):3S-6S.	No handbook
42	Mozes B, Shani M. **Clinical guidelines. Background, methodology and significance.** *Harefuah* 1993,**124**(10):634-7.	Unable to retrieve
43	National Federation of Cancer Centres. **Guide méthodologique 2005 pour la definition, la mise en oeuvre, et la realisation pratique d´une veille scientifique destine a identifier la nécessité de mise a jour de Recommandations pour la pratique clinique.** Paris: UNICANCER 2005.	No updating guidance
44	National Health and Medical Research Council. **Procedures and requirements for meeting the 2011 NHMRC standard for clinical practice guidelines (CP 133, 133A).** Canberra: National Health and Medical Research Council 2011.	No handbook
45	New Zealand Guidelines Group. **New Zealand Guidelines Group – Grading System for Guidelines.** Wellington: New Zealand Guidelines Group 2004.	No handbook
46	New Zealand Guidelines Group. **Tools for Guideline Development and Evaluation.** Wellington: New Zealand Guidelines Group 2003.	No updating guidance
47	New Zealand Guidelines Group. **Glossary / Methodology Terms.** Wellington: New Zealand Guidelines Group 2003.	No handbook
48	Norwegian Directorate for Health. **Retningslinjer for retningslinjer.** Oslo: Norwegian Directorate for Health 2002.	No handbook
49	Palda VA, Davis D, Goldman J. **A guide to the Canadian Medical Association handbook on clinical practice guidelines.** *CMAJ*. 2007,**177**(10):1221-6.	No handbook
50	Ricci S, Celani MG, Righetti E. **Development of clinical guidelines: methodological and practical issues.** *Neurol Sci* 2006,**27**(3):S228-30.	No handbook
51	Rosenfeld RM, Shiffman RN. **Clinical Practice Guidelines: A Manual for Developing Evidence-Based Guidelines to Facilitate Performance Measurement and Quality Improvement.** *Otolaryngol Head Neck Surg* 2006,**135**(4):S1-28.	Older version of included handbook
52	Rosenfeld RM, Shiffman RN. **Clinical practice guideline development manual: a quality-driven approach for translating evidence into action.** *Otolaryngol Head Neck Surg.* 2009,**140**(6):S1-43.	Older version of included handbook
53	Schünemann HJ, Munger H, Brower S, O'Donnell M, Crowther M, Cook D, Guyatt G. **Methodology for guideline development for the Seventh American College of Chest Physicians Conference on Antithrombotic and Thrombolytic Therapy: the Seventh ACCP Conference on Antithrombotic and Thrombolytic Therapy.** *Chest* 2004,**126**(3):174S-178S.	No handbook
54	Scottish Intercollegiate Guidelines Network. **Continuing professional development. A manual for SIGN guideline developers.** Edinburgh: Scottish Intercollegiate Guidelines Network 2002.	No handbook

55	Scottish Intercollegiate Guidelines Network. **Guideline development in fifty easy steps [summary of the SIGN guideline development process].** Edinburgh: Scottish Intercollegiate Guidelines Network 2004.	No handbook
56	Soroka M, Barresi B, Oliver G, Perry C. **Guideline development process for optometric care of the patient with diabetes mellitus. American Optometric Association.** *J Am Optom Assoc* 1994,**65**(8):573-7.	No handbook
57	Stolberg HO. **The development of radiology guidelines in Canada, Part 2.** *Can Assoc Radiol J*. 1999,**50**(3):152-5.	Unable to retrieve
58	Swiss Medical Association. **Guideline für guidelines.** Bern: Swiss Medical Association 2000.	No updating guidance
59	Van Biesen W, van der Veer SN, Jager KJ, Fouque D, Wanner C, Vanholder R. **What guidelines should or should not be: implications for guideline production.** *Nephrol Dial Transplant* 2013,**28**(8):1980-4.	No handbook
