# Supplementary material for: Guidance for updating clinical practice guidelines: a systematic review of methodological handbooks
Source: Implement Sci. 2014 Jan 2;9:3. doi: 10.1186/1748-5908-9-3 (PMC3904688; doi:10.1186/1748-5908-9-3)
Supplement: Additional file 3 — Included handbooks [ordered by organisation]. [file 1748-5908-9-3-S3.pdf]

Additional file 3: Included handbooks [ordered by organisation]

Number	Organisation	Country	Title handbook	Year
1	U.S. Preventive Services Task Force[43]	United States of America	U.S. Preventive Services Task Force Procedure Manual.	2008
2	American Academy of Otolaryngology–Head and Neck Surgery[38]	United States of America	Clinical Practice Guideline Development Manual, Third Edition: a quality-driven approach for translating evidence into action.	2013
3	American College of Chest Physicians[51]	United States of America	Evidence-based Guideline Development Process	Not reported
4	American College of Occupational and Environmental Medicine[39]	United States of America	Methodology to Update the Practice Recommendations in the American College of Occupational and Environmental Medicine's Occupational Medicine Practice Guidelines, Second Edition.	2008
5	American College of Physicians[40]	United States of America	The development of clinical practice guidelines and guidance statements of the American College of Physicians: summary of methods.	2010
6	American Heart Association[44]	United States of America	Methodology Manual and Policies From the ACCF/AHA Task Force on Practice Guidelines.	2010
7	American Society of Clinical Oncology[37]	United States of America	American Society of Clinical Oncology Guideline Procedures Manual.	2011
8	American Urological Association[41]	United States of	Overview: Standard Operating Procedures.	2011

		America		
9	Bundesärztekammer [28]	Germany	National Disease Management Guidelines.	2002
10	Arzneimittelkommission der deutschen Ärzteschaft[27]	Germany	Leitfaden für die Erstellung von Therapieempfehlungen.	2011
11	Canadian Medical Association[46]	Canada	Handbook on Clinical Practice Guidelines.	2007
12	Canadian Thoracic Society[50]	Canada	Canadian Thoracic Society: Presenting a new process for clinical practice guideline production.	2009
13	Agència d'Avaluació de Tecnologia i Recerca Mèdiques.[25]	Spain	Guies de pràctica clínica.	2002
14	Domus Medical Flemish College of General Practitioners[34]	Belgium - Flanders	Algemeen Stramien voor de Ontwikkeling van Aanbevelingen van Goede Medische Praktijkvoering.	2007
15	Drug Commission of the German Medical Association[26]	Germany	Handbuch zur Entwicklung regionaler Leitlinien.	2006
16	Duodecim Finnish Medical Society[33]	Finland	Submitted NHS Evidence Accreditation Application.	2010
17	Duodecim Medical Publications[32]	Finland	Preface: What is Evidence-Based Medicine Guidelines.	2008
18	Kwaliteitsinstituut voor de Gezondheidszorg CBO[23]	The Netherlands	Evidence-based Richtlijnontwikkeling Handleiding voor werkgroepleden.	2007
19	European Region Of The World Confederation For Physical Therapy[24]	Europe	Framework for Clinical Guideline Development in Physiotherapy.	2004
20	European Society of Gastrointestinal Endoscopy[29]	Europe	European Society of Gastrointestinal Endoscopy (ESGE) Guideline Development	2012

			Policy.	
21	Ärztliche Zentralstelle Qualitätssicherung[31]	Germany	National Disease Management Guidelines: Method Report.	2002
22	Centrul Naţional de Studii Medicina Familiei[22]	Romania	Metodologie elaborarii ghidului de practica.	Not reported
23	Working Group on CPG Updates[5]	Spain	Updating Clinical Practice Guidelines in the Spanish National Health System: Methodology Handbook.	2009
24	Guidelines and Protocols Advisory Committee[42]	Canada	GPAC Handbook	2011
25	Haute Autorité de Santé[21]	France	Élaboration de recommandations de bonne pratique.	2010
26	Italian Society for Haemostasis and Thrombosis[30]	Italy	Objectives and methodology: Guidelines of the Italian Society for Haemostasis and Thrombosis (SISET).	2009
27	Joanna Briggs Institute Synthesis Science Unit[45]	International	Best Practice Information Sheet (BPIS) Procedures.	Not reported
28	National Health and Medical Research Council[36]	Australia	A guide to the development, implementation and evaluation of clinical practice guidelines.	1998
26	National Institute for Health and Clinical Excellence[6]	United Kingdom	The guidelines manual.	2012
30	New Zealand Guidelines Group[49]	New Zealand	Handbook for the Preparation of Explicit Evidence-base Clinical Practice Guidelines.	2001
31	Scottish Intercollegiate Guidelines Network[14]	United Kingdom – Scotland	SIGN 50: A guideline developer's handbook.	2011

32	Society for Vascular Surgery[52]	United States of America	Guideline methodology of the Society for Vascular Surgery including the experience with the GRADE framework.	2011
33	Caring for Australasians with Renal Impairment[48]	Australia	A Guide for Writers.	2009
34	Therapeutic Guidelines Limited[47]	Australia	How Therapeutic Guidelines are produced.	2011
35	World Health Organization[35]	International	Guidelines for WHO guidelines.	2003
